# Accurate Single-Cell Clustering through Ensemble Similarity Learning

**DOI:** 10.3390/genes12111670

**Published:** 2021-10-22

**Authors:** Hyundoo Jeong, Sungtae Shin, Hong-Gi Yeom

**Affiliations:** 1 Department of Mechatronics Engineering, Incheon National University, Incheon 22012, Korea; hdj@inu.ac.kr; 2 Department of Mechanical Engineering, Dong-A University, Busan 49315, Korea; stshin@dau.ac.kr; 3 Department of Electronics Engineering, Chosun University, Gwangju 61452, Korea

**Keywords:** single-cell RNA sequencing, zero-inflated noise reduction, ensemble similarity estimation, correspondence network, visualization and clustering, imputation

## Abstract

Single-cell sequencing provides novel means to interpret the transcriptomic profiles of individual cells. To obtain in-depth analysis of single-cell sequencing, it requires effective computational methods to accurately predict single-cell clusters because single-cell sequencing techniques only provide the transcriptomic profiles of each cell. Although an accurate estimation of the cell-to-cell similarity is an essential first step to derive reliable single-cell clustering results, it is challenging to obtain the accurate similarity measurement because it highly depends on a selection of genes for similarity evaluations and the optimal set of genes for the accurate similarity estimation is typically unknown. Moreover, due to technical limitations, single-cell sequencing includes a larger number of artificial zeros, and the technical noise makes it difficult to develop effective single-cell clustering algorithms. Here, we describe a novel single-cell clustering algorithm that can accurately predict single-cell clusters in large-scale single-cell sequencing by effectively reducing the zero-inflated noise and accurately estimating the cell-to-cell similarities. First, we construct an ensemble similarity network based on different similarity estimates, and reduce the artificial noise using a random walk with restart framework. Finally, starting from a larger number small size but highly consistent clusters, we iteratively merge a pair of clusters with the maximum similarities until it reaches the predicted number of clusters. Extensive performance evaluation shows that the proposed single-cell clustering algorithm can yield the accurate single-cell clustering results and it can help deciphering the key messages underlying complex biological mechanisms.

## 1. Introduction

Single-cell sequencing technologies have been gaining popularity and importance in biomedical research because it can provide effective means to obtain gene expression profiles for hundreds of thousands cells in a high-throughput manner [1,2,3,4], i.e., although a next-generation sequencing can measure an average gene expression for numerous cells in the tissues, the single-cell sequencing enables the profiling of the gene expressions for individual cells. Due to the distinct advantages of the single-cell sequencing, it can accelerate a development of novel drugs and effective therapeutic methods for complex diseases such as cancers and neurodegenerative diseases by providing a breakthrough to unveil and interpret key messages underlying complex biological mechanisms in different cell types.

Single-cell clustering is an essential first step of single-cell sequencing for a downstream analysis because single-cell sequencing techniques typically do not provide auxiliary information such as cell types for each gene expression value and their spatial origins in tissue samples [5]. Once we obtain an accurate identification of single-cell clusters, it can help identifying cell types and marker genes that can play a pivotal role in elucidating the biological insights from biological experiments. However, since the single-cell sequencing protocols employ relatively the small amount of mRNA samples to read the gene expression profile for each cell, the true gene expression may not be detected and the missing information results in excessive zeros in the sequencing results, where it can be modeled as a zero-inflated noise. The technical noise makes it difficult to develop effective single-cell clustering algorithms.

Several single-cell clustering methods have been proposed based on different strategies and distinctive strengths. For example, pcaReduce [6] obtain the low-dimensional feature vector for each cell through PCA (principal component analysis), and it makes the initial clusters by the *K*-means clustering algorithm based on the principal components. Next, it computes the probability density functions for each pair of clusters. Then, based on the probability density functions, pcaReduce iteratively combines a pair of clusters with the highest probability until it reaches the user-defined number of clusters. TSCAN [7] first estimates the similarities between cells through the Euclidean distance and it also determines the complete linkage based on gene expression patterns of single cells. Then, it yields the single-cell clustering through a hierarchical clustering. scCLUE [8] first constructs an ensemble similarity network by integrating multiple similarity networks that can represent the cell-to-cell similarities through different similarity estimates. Then, it yields the accurate and consistent single-cell clusters through the Louvain algorithm [9]. Although the aforementioned algorithms can lead to the accurate prediction of the single-cell clustering, it requires the true number of clusters as a user input parameter, where it is typically unknown. To resolve the problem, single-cell clustering algorithms, where it can automatically determine the number of clusters in the single-cell sequencing data, have been introduced. Seurat [10] adopts a network-based clustering framework, where it is also employed in other algorithms [11,12]. Seurat first reduces the dimension of the single-cell sequencing through PCA and it determines the similarities between cells based on the first 10 PCs (principal components). Next, it constructs a KNN (*K*-nearest neighbors) network based on the estimated similarities. Finally, it identifies the single-cell clusters through the Louvain algorithm. SIMLR [13] derives a robust estimation of the cell-to-cell similarity based on the multiple Gaussian kernels with different parameters. Based on the ensemble learning for the cell-to-cell similarity, SIMLR determines the single-cell clusters through the *K*-means clustering algorithm. To the best of our knowledge, CIDR [14] is the first single-cell clustering algorithm that takes the technical noise into account to derive a reliable single-cell clustering. First, it reduces the zero-inflated noise in a single-cell sequencing data through an implicit imputation method. Then, CIDR obtains the single-cell clusters through a hierarchical clustering algorithm based on the dissimilarity between each cell. SC3 [15] constructs the cell-to-cell similarity matrix based on the Euclidean distance or correlation between cells. Next, it changes the similarity matrix through PCA or normalized Laplacian. Then, based on the transformed similarity matrix, it determines the single-cell clustering through a hierarchical clustering algorithm. SinNLRR [16] adopts subspace clustering approach with low-rank representation to derive accurate estimation of cell-to-cell similarity. It assumes that the gene expression of one cell can be represented as the combination of gene expressions of cells in the same type. Based on this assumption, it constructs the optimization problem with low-rank and non-negative constraints. Then, it solves the optimization problem through an alternating direction method of multipliers and obtains a robust clustering through spectral clustering. RGGC [17] also adopts a subspace clustering method to derive robust single-cell clustering, where it assumes that each cell can be represented the linear combination of cells in the same subspace. Then, it employs a regularized Gaussian graphical model to construct the optimization problem with $L_{2}$ regularization. Finally, it yields a single-cell clustering through Louvain algorithm. One distinctive advantage of RGGC is that there is no free parameter so that it can save a time for parameter tuning.

In this paper, we propose an accurate single-cell clustering algorithm called SICLEN (SIngle-cell CLustering based on effective noise reduction through ENsemble similarity network). First, to avoid the optimal feature gene selection problem, we identify a set of potential feature genes that can have a high probability to be marker genes for each cell type. Second, we represent a cell-to-cell similarity through an ensemble similarity network, i.e., we consider a cell as a node and connect two cells by inserting an edge if a pair of cells show consistently high similarity based on the multiple similarity estimates that can be obtained by the different subsamples of the potential feature genes. Then, we effectively reduce a zero-inflated noise through a random walk with restart over the ensemble similarity network and predict the number of clusters using Rubin index [18]. Finally, starting from a large number of small size but highly coherent single-cell clusters that can be obtained through the *K*-means clustering algorithm, we obtain an accurate clustering by iteratively merging a pair of clusters if they record the highest similarity among the collection of clusters until it reaches the predicted number of clusters. Based on the comprehensive evaluations, we demonstrate that the proposed single-cell clustering algorithm can achieve accurate and reliable clustering results in terms of algorithmic and biological perspectives.

## 2. Materials and Methods

### 2.1. Datasets

We employed 12 single-cell sequencing datasets to evaluate the performance of single-cell clustering algorithms. To obtain the single-cell sequencing datasets, we accessed the Gene Expression Omnibus (GEO) provided by NCBI (National Center for Biotechnology Information) and searched the accession number in Table 1. Then, we downloaded a raw count (or gene expression) matrix for each data. Finally, we obtained the processed gene expression matrix after removing genes that are not expressed across cells because these genes are not informative to derive clustering results. Darmanis et al. provided a gene expression profile for cells in a human brain [19]. Among nine cell types, we only excluded the cells annotated by ‘hybrid’ because these cells can be considered to be an intermediate type between neurons and astrocytes [20]. Usoskin et al. obtained a single-cell sequencing data from mouse sensory neurons, and it includes a gene expression profile for peptidergic nociceptors (PEP), non-peptidergic nociceptors (NP), neurofilament containing (NF), and tyrosine hydroxylase containing (TH) [21]. Kolodziejczyk et al. provided a single-cell sequencing data for pluripotent cells under different environmental conditions [22]. Romanov et al. provided a single-cell sequencing data for a hypothalamus in a mouse brain and it includes seven major cell types such as oligodendrocytes, astrocytes, ependymal cells, microglial cells, endothelial cells, vascular and smooth muscle lineage cells, and neurons [23]. Xin et al. provided a gene expression profile for cells in a human pancreatic islet [24]. It provides alpha, beta, delta, and gamma cells from non-diabetic and T2D (type 2 diabetes) organ donors. Klein et al. provided a single-cell sequencing data for mouse embryo stem cells [2]. Braon et al. obtained gene expression profiles for cells in human and mouse pancreatic islets from four human donors and two mice strains [25]. In these datasets, we only kept the following cell types: alpha, beta, delta, ductal, gamma, and acinar because the number of the other types is much smaller than the major cell types. Additionally, we also removed acinar cells for mouse datasets for the same reason.

### 2.2. Parameter Settings for Each Algorithm

We compared the performance of the proposed single-cell clustering algorithm with the state-of-the-art methods: Seurat [10], SIMLR [13], CIDR [14], and SC3 [15]. We also compared the proposed method with the clustering results through t-SNE [26] followed by the *K*-means clustering algorithm because it is one of the popular approaches to quickly obtain the naive clustering results. We obtained clustering results for each algorithm based on the R implementation with the default parameter settings. Moreover, to determine the number of clusters for each algorithm, we used the recommended method or internal function for each algorithm so that the number of predicted clusters for each method can be different. Please note that we only used the true number of clusters for t-SNE followed by *K*-means clustering algorithm. We performed all experiments on a Linux (Ubuntu 18.04.4) server with Intel Xeon processor (2.4 GHz) with 24 cores and 256 GB memory.

### 2.3. Motivation and Overview of the Proposed Method

To obtain a reliable single-cell clustering result, accurately estimating a cell-to-cell similarity is a crucial first step. However, from a practical point of view, there are several hurdles to derive a reliable estimation of the cell-to-cell similarity. First, since a single-cell sequencing can simultaneously profile the gene expression levels for hundreds of thousands of cells, each cell can be represented as a high-dimensional vector. It is computationally intensive to evaluate the pairwise similarities for high-dimensional vectors representing the gene expression characteristics of numerous cells. Second, due to the technical limitations, single-cell sequencing data includes a larger number of artificial zeros called dropouts and the zero-inflated noise makes the problem challenging to derive a reliable estimation of a cell-to-cell similarity. Furthermore, it is also demanding to select a set of the optimal genes that can yield a reliable single-cell clustering in terms of the mathematical and biological perspectives.

**Figure 1:**
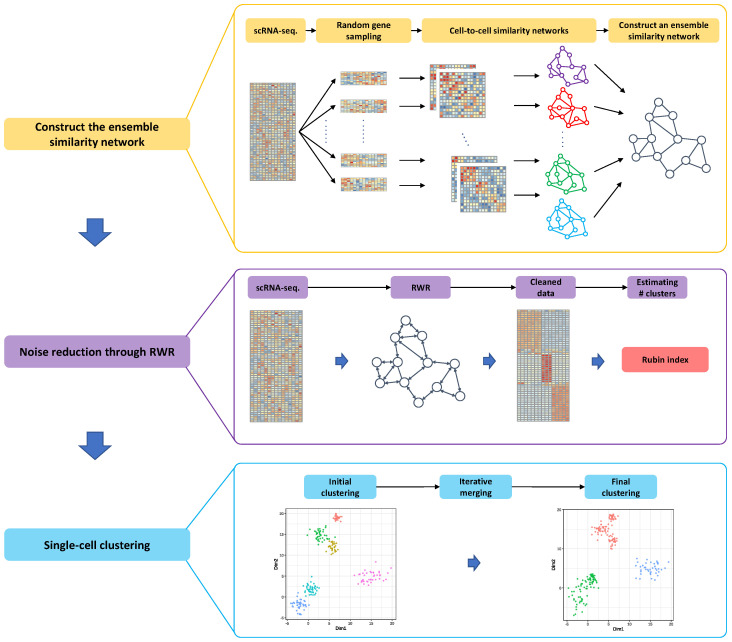
Graphical overview of the proposed single-cell clustering algorithm. Please note that the illustrations in a highlighted box are a toy example for each step.

To lower these hurdles, we propose a novel method to reliably estimate a cell-to-cell similarity through an ensemble feature selection and the effective noise reduction based on a random walk with restart framework. Although a single-cell sequencing includes a larger number of genes and cells, each cell type generally has distinctive marker genes that can be highly expressed only in a particular cell type. Hence, if we accurately identify the marker genes for each cell type of interest, we can dramatically enhance an accuracy of clustering results and reduce a dimensionality of a single-cell sequencing data, where it can consequently reduce a computational complexity of single-cell clustering algorithms. However, it is practically infeasible to determine the optimal marker (or feature) genes because of the high dimensionality of a single-cell sequencing data. Additionally, it is also challenging to define an effective objective function to select the effective feature genes for single-cell clustering algorithms in terms of the biological and mathematical perspectives. To avoid the optimal feature gene selection problem, we first select a set of potential marker genes and we estimate multiple cell-to-cell similarities based on the different subsets of the potential marker genes, where it can be obtained through the random gene sampling. Through the multiple estimations of the cell-to-cell similarity based on the different sets of genes, if two cells achieve consistently high similarity, we consider that these cells are highly likely to be classified into the same cell type. Although SC3 exploits different similarity measurements using Euclidean distance, Pearson and Spearman correlation, it only considers a single set of genes to determine the similarity estimates. However, the proposed method employs multiple sets of genes to derive the similarity measurements and integrates these metrics to yield the robust cell-to-cell similarity, where it is key difference between the proposed method and SC3. Based on the ensemble similarity measurements, we can construct the ensemble similarity network, where a cell can be modeled as a node and their similarities can be described as an edge. For more details, as we can see in a toy example in Figure 1, we can have various similarity measurements based on the different subsets of feature genes that can be obtained through a random gene sampling. The diverse similarity measurements can yield different network topology that can represent the similarity among numerous cells in different perspectives so that it helps in identifying the cells achieving a consistently high similarity. Next, we can reduce the zero-inflated noise through the wisdom of the crowd, i.e., since the cells in the same cell type typically show the similar gene expression patterns, although there is a missing gene expression value in one cell because of dropout events, the gene expressions can be clearly captured in the other cells in the same type. Hence, we can employ the gene expression patterns from the neighboring nodes (i.e., cells) in the ensemble similarity network to infer the missing gene expression values (For details, see Section 2.6 and Equation (6)). After reducing the technical noise, we first predict a larger number of small size but highly coherent clusters using the cleaned single-cell sequencing data. Then, we continuously merge a pair of clusters if they show the largest similarity among clusters until we reach the reliable clustering results. Based on the above motivation, the proposed method consists of three major steps: (i) constructing the ensemble similarity network based on the similarity estimations under different conditions (i.e., feature gene selections), (ii) reducing the artificial noise through a random walk with restart over the ensemble similarity network, and (iii) performing an effective single-cell clustering based on the cleaned gene expression data.

### 2.4. Data Normalization

Suppose that we have a single-cell sequencing data and it provides gene expression profiles as the *M* by *N*-dimensional matrix Z, where *M* is the number of genes and *N* is the number of cells. Please note that the proposed method can accept non-negative value (e.g., read counts) as a gene expression profile if it represents the relative expression levels of each gene. Since cells in a single-cell sequencing typically have different library sizes, we have normalized the gene expression profile through the counts per million (cpm) to alleviate an artificial bias induced by the different library sizes. Then, similarly to other single-cell clustering algorithms [10,13,14,15], we also take a log-transformation because relative gene expression patterns may not be clearly captured if a single-cell sequencing data includes the extremely large numeric values and the concave functions such as a logarithmic function can effectively scale down the extremely large values into a moderate range. The normalized gene expression profile X is given by

(1) $$X=\log_{2}\left(1+\mathrm{cpm}\left(Z\right)\right),$$

where cpm(·) is a function to normalize the library size through the counts per million.

### 2.5. Ensemble Similarity Network Construction

We employ a graphical representation of a single-cell sequencing data in order to describe the cell-to-cell similarity that can yield an accurate single-cell clustering because a graph (or network) can provide a compact representation of complex relations between multiple objects, i.e., we construct the cell-to-cell similarity network $G=\left(V,E\right)$, where a node $v_{i}\in V$ indicates *i*-th cell and an edge $e_{i,j}\in E$ represents the similarity between the *i*-th and *j*-th cells. Suppose that the weight of an edge $e_{i,j}$ is proportional to the similarity of cells so that cells with the larger similarity can have the higher edge weight.

To begin with, given a normalized single-cell sequencing data X, we identify a set of potential feature genes F, where it is a collection of marker gene candidates for each cell type. However, if we do not have a biological prior knowledge, it is challenging to collect reliable marker gene candidates for each cell type in a practical point of view. Moreover, although we have a domain knowledge for marker genes for each cell type, it is also possible that the novel marker genes may not be considered to predict single-cell clusters and this missing information can decrease an accuracy of single-cell clustering results. To make a reliable collection of marker gene candidates without a biological prior knowledge, we take the typical properties of marker genes into account. That is, since the marker genes are generally highly expressed in a specific cell type and rarely expressed in the rest of cells, we hypothesize that the marker gene candidates have the following properties: (i) the marker gene candidates are highly expressed so that they also show relatively higher mean expression values and (ii) the variance of the marker gene candidates across cells is relatively high. Based on the assumption, we collect the genes with relatively higher mean and larger variance across cells and define these genes as the set F of the potential feature genes. To this aim, we calculate the row-wise mean and variance of the normalized gene expression matrix X. Then, we select genes whose mean expression level is greater than the median of the expected gene expression values. Among these genes, we only retain top *K* percent genes with the largest variance. Please note that in this study, we select the top five percent of genes to make the set F of potential feature genes.

Next, to construct the ensemble similarity network $G_{E}$, we consider each cell as a node and insert an edge between cells if their similarity is greater than a threshold, i.e., in order to accurately represent the cell-to-cell correspondence as an ensemble similarity network, we obtain multiple similarity measurements based on the different feature sets and construct the ensemble similarity network by inserting edges between nodes (i.e., cells) if they show consistently high similarity scores for multiple similarity evaluations. Since different feature sets can yield different similarity estimations, we can identify cells that can achieve consistently high similarity through various similarity estimates based on the random gene sampling. First, we obtain a subset of potential feature genes $f^{l}\subset F$ through a *l*-th random gene sampling, where it follows a uniform distribution, i.e., each gene in the set **F** can have an equal probability to be sampled so that multiple similarity estimations based on the different gene sampling can increase a diversity of similarity measurements. Next, we reduce the dimensionality of a single-cell sequencing data through PCA and evaluate the cell-to-cell similarity using Pearson correlation based on the first 10 PCs (principal components). Please note that although it can be freely adjusted depending on the experimental environments, since the explained variance using the first 10 PCS can cover more than 80% of total variance for each data, we employ the first 10 PCs for the default setting in the proposed method. Then, based on the estimated similarity (i.e., Pearson correlation between cells), we construct a KNN (*K*-nearest neighbors) network for the *l*-th feature sampling by inserting up to *K* edges for each cell so that they can have the *K* neighboring cells with the largest similarity. Please note that based on the empirical experiments, we set the default parameter of *K* as 30. Although all nodes in the KNN network can have up to *K* neighbors, when considering a profiling capability of single-cell sequencing techniques, it is completely possible that there are a larger number of cells in the same type so that using only *K* neighbors would not be effective approach to represent the cell-to-cell similarity. To maximize the benefits of the wisdom of the crowd, it is desirable to introduce a greater number of edges that can correctly connect cells in the same type. In fact, if we can identify the perfect similarity network, it is a collection of cliques (i.e., an induced subgraph with every possible edge), but the perfect similarity network is typically unknown. Given the KNN network for the *l*-th similarity measurement, a feasible solution to induce a greater number of edges in the KNN network is introducing a new edge $e_{i,j}^{l}$ if the correlation between *i*-th and *j*-th cells is greater than the threshold $e_{th}^{l}$. To determine the threshold $e_{th}^{l}$, where it can take a reasonable cell-to-cell similarity level into account, we make a set of edge weights for the *l*-th KNN network (i.e., $W_{l}=\left\{c_{i,j}^{l}|e_{i,j}>0\right\}$, where $c_{i,j}^{l}$ is Pearson correlation between *i*-th and *j*-th cells based on the *l*-th similarity estimate). Then, we decide the threshold $e_{th}^{l}$ as the tenth percentile of the Pearson correlations in the set $W_{l}$. Finally, we can induce new edges $e_{th}^{l}$ if Pearson correlation between *i*-th and *j*-th cell is greater than the threshold $e_{th}^{l}$. Based on the *l*-th similarity evaluation, the adjacency matrix for the updated KNN network is given by

(2) $$A^{l}\left[i,j\right]=\left\{\begin{array}{cc} 1, & if\;\;\;c_{i,j}^{l}>e_{th}^{l}\\ 0, & o.w. \end{array}\right.$$

Through the multiple similarity measurements, we have *L* adjacency matrices and the ensemble similarity network $G_{E}$ can be obtained by integrating these adjacency matrices. The adjacency matrix for the ensemble similarity network $G_{E}$ is given by

(3) $$A_{E}=\sum_{l=l}^{L}A^{l},$$

where $A^{l}$ is the adjacency matrix for updated KNN network based on the *l*-th similarity estimation.

Although the ensemble similarity network $G_{E}$ can provide a robust description for the cell-to-cell similarity, it still has a probability to include false edges by chance, where it connects the cells that can be classified in different types if the random gene sampling unexpectedly results incorrect similarity assessment. However, since it is difficult to decide indeed false edges, we remove all edges in the ensemble similarity network if their weights are smaller than $\frac{L}{2}$, where *L* is the number of similarity measurements, because their edge weights show a high similarity less than 50% for overall estimates. Please note that we use the 20 similarity measurements for a default parameter. After removing these unreliable edges, we obtain a refined adjacency matrix ${\tilde{A}}_{E}$. To obtain the transition probability of a random walker, we normalize the adjacency matrix ${\tilde{A}}_{E}$ to make it as a legitimate stochastic matrix, where the transition probability matrix is given by

(4) $$P_{E}={\tilde{A}}_{E}\times D,$$

where D is a $\left|M\right|\times \left|M\right|$-dimensional diagonal matrix such that $D\left[i,j\right]=\sum_{\forall j}^{}{\tilde{A}}_{E}\left[i,j\right]$. Moreover, since biological networks typically tend to make a dense connection if two nodes are in the same community, the neighbors of the neighbors can have a high possibility to be in a same community and we also take the second order structure of the network into account [27,28]. If we consider higher order network structure, we can make a denser network by inducing a greater number of edges. However, since it can also induce a greater number of false positive edges that can connect two nodes with different labels, we only consider the second order structure of the network. Since the random walker on the network can reach neighbors of the neighbors through the two-hops, the final transition probability matrix of the random walker over the ensemble cell-to-cell similarity network is given by

(5) $$R=P_{E}\times P_{E}.$$

### 2.6. Reducing Zero-Inflated Noise in a Single-Cell Sequencing Data

Once we have the reliable ensemble similarity network that can effectively represent the cell-to-cell correspondence, we can reduce the zero-inflated noise in a single-cell sequencing data to enhance an accuracy of the clustering results. Since a gene expression profile in the same cell type typically shows the similar patterns, we can leverage the gene expression patterns of neighboring cells in the learned ensemble similarity network to reduce the zero-inflated noise, i.e., although there is a missing gene expression in one cell, it can be captured by the other cells with the same type (i.e., neighboring nodes in the ensemble similarity network). Therefore, the missing gene expression patterns can be recovered by employing the information from the neighboring nodes. Based on the hypothesis, to infer the missing gene expression values, we adopt a random walk with restart approach over the ensemble similarity network because a random walk with restart framework can effectively diffuse the gene expression values to its neighboring nodes. Hence, we can obtain the averaged gene expression of the neighboring cells in the ensemble similarity network by examining a long-term behavior of the random walker. Additionally, we can obtain the balanced integration between the gene expression of one cell and the averaged gene expression from the neighboring nodes by adjusting a restarting probability of the random walker. Given a transition probability matrix of the random walker, the long-term characteristic of the random walker can be derived by the following equation:

(6) $$r=\alpha \cdot R\cdot r+\left(1-\alpha \right)\cdot e,$$

where α is a weighting coefficient that can adjust the restarting probability of the random walker, and r is a steady-state probability of the random walker that can indicate the long-run behavior of the random walker, and e is the initial probability distribution that can determine the restart location of the random walker. Please note that we exploit the log-transformed normalized gene expression as the initial probability distribution of the random walker and the restarting probability of the random walker is set to 0.7 as a default setting. Since the steady-state probability r indicates the long-run behavior of the random walker, it can be interpreted as the balanced sum of the gene expression for one cell and the expected gene expression values from its neighboring nodes, where it is a noise reduced single-cell sequencing data. The steady-state probability r can be obtained by solving following linear equation:

(7) A·r=b,

where A is $\left(I-\alpha \cdot W\right)$ and b is $\left(1-\alpha \right)\cdot e$, respectively.

### 2.7. Method to Estimate the True Number of Clusters

To obtain an accurate single-cell clustering, we need to estimate the number of clusters (i.e., cell types) in a single-cell sequencing data because the true number of cell types is typically unknown. Since the zero-inflated noise can be reduced through the random walk with restart technique, we use the cleaned data to estimate the true number of clusters. First, we have normalized the cleaned single-cell sequencing data because the denoising process through the random walk with restart can yield different library sizes, where it can result undesirable bias of the relative gene expressions across different cells. Next, to reduce a computational complexity and capture the accurate estimation results, we identify the feature genes with larger mean and variance, and obtain the low-dimensional representation through PCA. Based on the first 10 PCs (principal components), we decide the number of clusters through Rubin index [18], where it is implemented in the R package called NbClust [29]. Please note that based on our experimental results, we empirically employ 10 principal components and Rubin index because including a greater number of principal components would not lead to an improved estimating accuracy and the combination achieves the best performance in our study.

### 2.8. Effective Single-Cell Clustering Algorithm through the Ensemble Similarity Network

To obtain an accurate single-cell clustering, we first start from a larger number of small size but highly consistent clusters and combine two clusters if they show the highest possibility to be the same cluster. Then, we proceed an iterative cluster merging step until the number of clusters reaches the estimated number of clusters. To this aim, we identify the feature genes based on the same approach for the ensemble network construction. Please note that we exploit one percent of genes for the feature genes. Then, we obtain the initial clusters through the *K*-means clustering algorithm because it can easily adjust the number of clusters. Moreover, since the random walk with restart framework can reduce the zero-inflated noise in a single-cell sequencing data, it can decrease a distance between cells in the same type and separate a distance between different cell types. Additionally, since the *K*-means clustering algorithm guarantees the convergence, we exploit the *K*-means clustering algorithm as a base framework. Please note that we employ 10 PCs and identify 20 initial clusters because we suppose that the single-cell sequencing datasets mostly have up to 20 cell types. However, these parameters can be adjusted depending on the user preferences. Since the initial clustering results can have singleton clusters, where it only has a single object (i.e., cell) in the cluster, we first merge these singleton clusters to another cluster that can achieve the largest possibility to be the same cell type, i.e., we compare the Pearson correlation between the gene expression of the cell in the singleton cluster and the mean expression of the feature genes for each cluster. Then, we combine the singleton node to the cluster that can achieve the highest Pearson correlation. Once we remove all singleton nodes, we continue the iterative merging process by comparing the Pearson correlation of the mean expression values for the feature genes in each cluster until the number of clusters is equal to the number of estimated clusters. Algorithm 1 provides the pseudo-code of the proposed single-cell clustering method. Supplementary Materials S1 provides the sensitivity analysis of SICLEN for different parameter settings.

**Algorithm 1:** Proposed single-cell clustering algorithm.

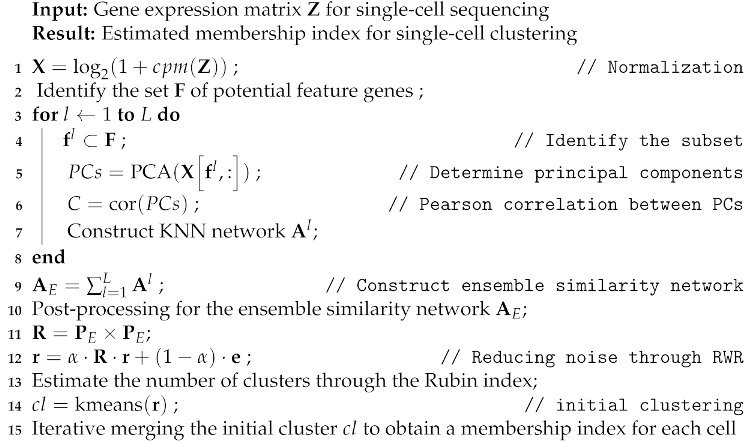



### 2.9. Performance Assessment Metrics

We evaluated the performance of the single-cell clustering algorithms based on the following perspectives: (i) algorithmic strength and (ii) biological relevance. To assess the single-cell clustering results in terms of the algorithmic point of view, we used the external information such as true labels for each cell and computed the following metrics: JCCI (Jaccard index), ARI (adjusted rand index), and NMI (normalized mutual information). Please note that higher JCCI, ARI, and NMI generally indicate an improved quality of the clustering results. To determine the performance metrics, we employed the R package called ClusterR [30].

Given *N* cells in a single-cell sequencing data, suppose that we have a collection of the true cell-type labels for each cell and the predicted cell-type labels (i.e., clustering labels) produced by each clustering algorithm, where the set of true cell-type labels is given by $C=\left\{c_{1},c_{2},\ldots ,c_{J}\right\}$ and the set of predicted clustering labels is given by $P=\left\{p_{1},p_{2},\ldots ,p_{K}\right\}$, respectively. Then, the Jaccard index (JCCI) is given by

(8) $$\mathrm{JCCI}\left(P,C\right)=\frac{TP}{TP+FP+FN},$$

where TP is the number of correct predictions (i.e., correctly clustered cells), and FP is the number of clustered cells with different true cell-type labels, and FN is the number of cells divided to the different clusters but they have the same true cell-type label.

Next, the adjusted rand index is given by

(9) $$\mathrm{ARI}\left(P,C\right)=\frac{\sum_{ij}\left(\frac{n_{ij}}{2}\right)-\left[\sum_{i=1}^{P}\left(\frac{a_{i}}{2}\right)\sum_{j=1}^{K}\left(\frac{b_{j}}{2}\right)\right]/\left(\frac{n}{2}\right)}{\frac{1}{2}\left[\sum_{i=1}^{P}\left(\frac{a_{i}}{2}\right)+\sum_{j=1}^{K}\left(\frac{b_{i}}{2}\right)\right]-\left[\sum_{i=1}^{P}\left(\frac{a_{i}}{2}\right)\sum_{j=1}^{K}\left(\frac{b_{j}}{2}\right)\right]/\left(\frac{n}{2}\right)},$$

where $n_{i,j}$ is the number of cells that have the *i*-th predicted label but it has the *j*-th true cell-type label, and $a_{i}$ and $b_{j}$ is the row and column sum of the contingency matrix (i.e., $a_{i}=\sum_{\forall j}n_{i,j}$ and $b_{j}=\sum_{\forall i}n_{i,j}$), respectively.

The normalized mutual information is given by

(10) $$\mathrm{NMI}\left(P,C\right)=\frac{2\cdot I(P,C)}{H(P)+H(C)},$$

where I(P,C) indicates the mutual information between P and C, and $H\left(\cdot \right)$ is the entropy of the clustering labels P and C.

Once we have the predicted clustering labels, a typical subsequent step for a downstream single-cell sequencing analysis is identifying the differentially expressed genes (DEGs), where it can be the marker genes for each cluster (i.e., cell type), because these DEGs can play an important role to design an accurate diagnosis and effective therapeutic methods for complex disease. To verify a biological relevance of a single-cell clustering, we identified the DEGs for each clusters based on the predicted clustering labels and compared it with the DEGs that are identified through the true cell-type labels because, if the predicted clustering labels are highly coherent with the true cell-type labels, we supposed that the DEGs identified through the predicted clustering labels and the true cell-type labels are also consistent with each other. To verify the accordance of DEGs for the predicted and true cell-type labels, we first determined DEGs for each cell type based on the true cell-type labels. In this study, we used the R package called MAST [31] to decide DEGs for each cell type. After identifying all DEGs, we only retain DEGs whose *p*-value is smaller than 0.05 and its fold change is greater than 1.5. We supposed that the DEGs satisfying the above requirements can have a statistical significance and considered these genes as a ground truth. Please note that Table 2 shows the total number of DEGs for the ground truth and these DEGs for the performance assessment are different to the potential marker genes to construct the ensemble similarity network. Based on the same approach, we also identified the DEGs through the predicted clustering labels by each algorithm and evaluated the agreement of DEGs identified by the true and predicted labels based on the precision, recall, and F-scores.

The recall is given by

(11) $$\mathrm{Recall}=\frac{TP}{TP+FN},$$

where *FN* is the number of DEGs that are not detected by the predicted labels, but it is discovered the true labels.

The precision is given by

(12) $$\mathrm{Precision}=\frac{TP}{TP+FP},$$

where *TP* is the number of DEGs that are consistently detected through the true and predicted labels, and *FP* is the number of DEGs that are only identified through the predicted clustering labels and not detected by the true cell-type labels.

The F-score is a harmonic mean of the precision and recall, where it is given by

(13) $$\text{F-score}=\frac{2\cdot \mathrm{Recall}\cdot \mathrm{Precision}}{\mathrm{Recall}+\mathrm{Precision}}.$$

## 3. Results

### 3.1. Performance Assessment Based on the True Cell-Type Labels

The major goal of the single-cell clustering is making a consistent group of cells because current single-cell sequencing protocols cannot provide the auxiliary information such as cell types even though it can simultaneously detect the relative gene expressions for a larger number of cells. Since a prior knowledge for the true cell type can play a pivotal role in a comprehensive analysis of the single-cell sequencing such as pseudo-temporal ordering [32,33,34] and gene regulatory networks [35,36,37], it is important to develop the accurate computational methods to predict the groups of cells with consistent labels.

**Figure 2:**
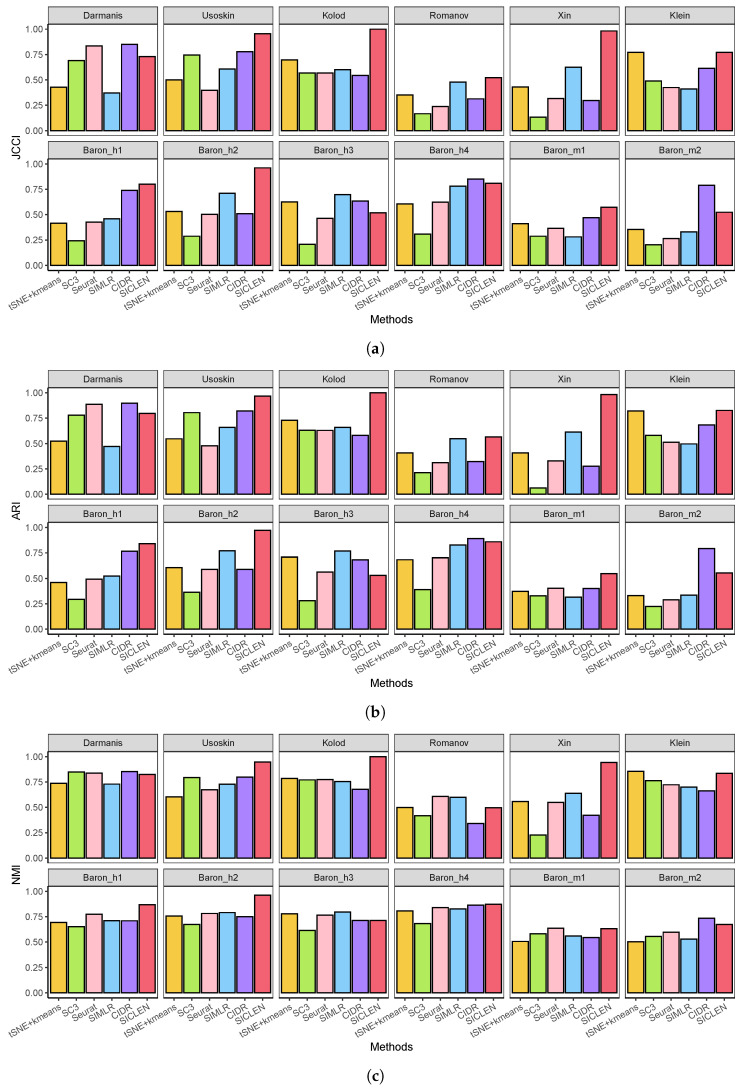
Performance metrics for different clustering algorithms. JCCI, ARI, and NMI are determined through the true cell-type labels. (**a**) Jaccard index for 12 single-cell sequencing datasets; (**b**) Adjusted rand index for 12 single-cell sequencing datasets; (**c**) Normalized mutual information for 12 single-cell sequencing datasets.

To evaluate a quality of clustering results, we compared the Jaccard index (JCCI) for each clustering algorithm because it can effectively assess the accuracy of clustering results by taking the size (i.e., the number of cells in each cluster) of each cluster into account. Based on the 12 single-cell sequencing datasets, SICLEN showed remarkably higher JCCI in Usoskin, Kolod., Xin, Klein, Baron_h1 and Baron_h2 datasets and it also achieved the comparable performance for other datasets (Figure 2a). Although CIDR achieved the next best JCCI scores for several datasets, SICLEN showed a clear gap to CIDR for most cases. Interestingly, the *K*-means clustering algorithm followed by t-SNE showed the comparable performance for other cutting-edge algorithms. One possible explanation is that although the t-SNE followed the *K*-means clustering algorithm employed the true number of clusters, each clustering algorithm used the predicted number of clusters based on their own strategies and it is possible that the algorithms are using the incorrect prediction for the number of clusters so that it results a severe deterioration the performance of clustering results. These results showed the importance of the method to predict the number of clusters in the single-cell sequencing data and we will discuss it in the following subsection. Next, although JCCI can capture the size factor for each clustering result, one drawback of the JCCI is that it does not take the true negatives into account. To assess the performance of the clustering algorithms in different perspectives, we also evaluated the adjusted rand index (ARI) for each clustering result to prove the effectiveness of the proposed method. In fact, ARI showed similar patterns to the JCCI for each clustering algorithm (Figure 2b). For example, although CIDR and SIMLR achieved the best ARI scores for the Darmanis and Baron_h4 datasets, the performance gap between the SICLEN and the best algorithm is negligible. However, when SICLEN attained the best performance in other datasets such as Kolod., Baron_h2, and Xin, it showed a clearly larger gap for the other competing algorithms. Finally, although the most algorithms showed the similar NMI scores, SICLEN still achieved distinctively higher NMI scores for most datasets such as Usoskin, Koloe., Xin, Klein, Baron_h1, and Baron_h2 datasets. Overall, based on the different performance metrics and datasets, we verified that SICLEN clearly outperformed the other single-cell clustering algorithms, and these results indicate that SICLEN can yield the consistent and accurate clustering results in terms of the algorithm perspectives.

### 3.2. Accurate Estimation of the True Number of Clusters through an Effective Noise Reduction

The accuracy of the single-cell clustering can be highly vulnerable to the following factors: (i) an accurate estimation of the cell-to-cell similarity, (ii) a tailored clustering method for the estimated similarity, (iii) a precise estimation of the correct number of clusters. However, it is easy to overlook the importance of the accurate estimation for the true number of clusters. As we can see in the previous subsection, if we adopt the incorrect number of clusters, the sophisticated method such as SC3 showed the inferior single-cell clustering results compared to the *K*-means clustering algorithm followed by t-SNE.

**Figure 3:**
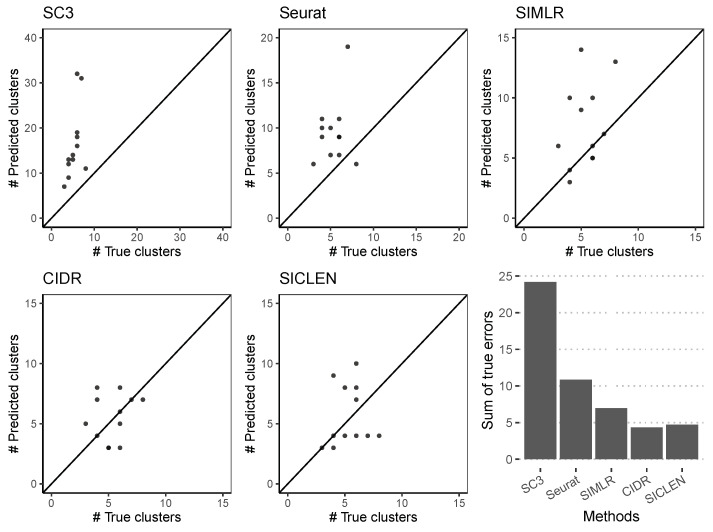
Comparison of the true number of clusters and predicted number of clusters for 12 datasets. Sum of errors can be determined by $\sum_{\forall i}^{}\frac{\left|J_{i}-K_{i}\right|}{\left|J_{i}\right|}$, where $J_{i}$ is the true number of clusters for *i*-th data and $K_{i}$ is the predicted number of clusters for *i*-th data.

To evaluate the accuracy of the inferred number of clusters, we first compared the true and predicted number of clusters for 12 datasets (Figure 3). As we can see, SC3 typically tends to overestimate the number of clusters and we verified that overestimating the number of clusters can clearly decrease the accuracy of the clustering results. Although there is an exceptional result in Seurat, Seurat and SIMLR achieved similar accuracy if we exclude the extreme result predicted by Seurat. SICLEN showed the smaller deviation for the predicted number of clusters compared to the other algorithms. To quantitatively assess the capability to predict the true number of clusters, we evaluated the sum of the percentage of true error such that $\sum_{\forall i}^{}\frac{\left|J_{i}-K_{i}\right|}{\left|J_{i}\right|}$, where $J_{i}$ is the true number of clusters and $K_{i}$ is the inferred number of clusters for the *i*-th single-cell sequencing data, respectively. In fact, although CIDR showed the smallest errors and SICLEN resulted the next smallest errors, their performance gap is negligible, but SICLEN and CIDR showed clearly smaller error compared to the other single-cell clustering algorithms, i.e., SICLEN and CIDR achieved the smallest deviation between the true and predicted number of clusters. One reasonable explanation is that CIDR and SICLEN adopt the effective method to deal with the zero-inflated noise in a single-cell sequencing but the other methods do not consider the technical noise so that the inherent zero-inflated noise can lead to the inferior prediction results for the other algorithms. Overall, these results clearly support that SICLEN can accurately estimate the true number of clusters compared to the other algorithms, where it is essential process to yield a reliable clustering result, and it also addresses the importance of the noise reduction methods in developing single-cell clustering algorithms.

### 3.3. Precise Identification of Differentially Expressed Genes through an Accurate Clustering

Identifying differentially expressed genes (DEGs) is one of the core tasks in downstream single-cell analysis pipelines because DEGs is the essential information to decipher key messages underlying complex biological mechanisms. Moreover, it enables the development of novel drugs and effective therapeutic methods for complex diseases such as cancers and neurodegenerative diseases. Since DEGs can be identified by comparing two groups, the essential prior knowledge to determine DEGs is the accurate cell-type labels obtained by either biological experiments or computational methods such as single-cell clustering algorithms. To this end, we hypothesized that if the predicted clustering labels are well agreed with the true cell-type labels, DEGs identified by the predicted clustering labels show high agreements to the DEGs derived by the true cell-type labels. Then, we compared the agreement of DEGs identified by the identified by the predicted clustering labels and the true cell-type labels. Based on the experimental assumption, we compared the recall, precision, and F-scores of the DEGs identified by each single-cell clustering result.

**Figure 4:**
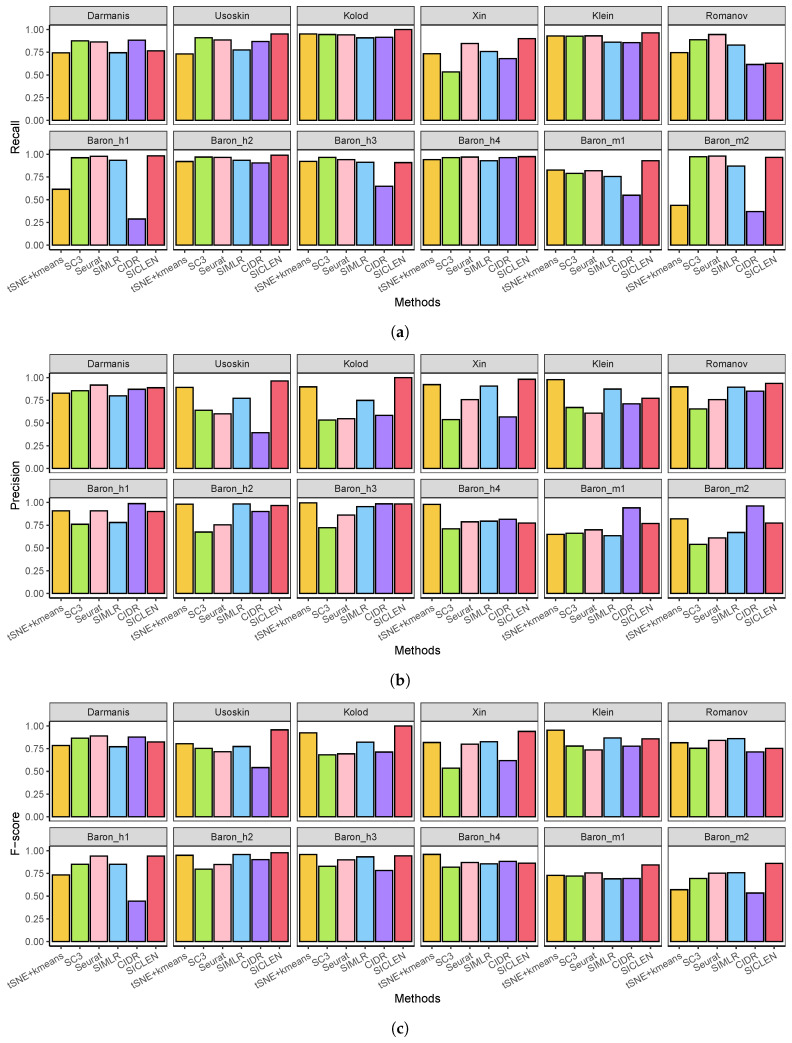
Precision, recall, and F-scores of the identification for DEGs through different clustering results. Please note that DEGs are identified through MAST [31] based on the true and predicted cell-type labels. (**a**) Recall of the identification for DEGs. Higher recall represents that a greater number of true DEGs are detected by group comparison; (**b**) Precision of the identification for DEGs. Higher precision indicates that there are smaller number of false DEGs in the prediction results; (**c**) F-scores of the identification for DEGs.

First, although SICLEN showed a higher recall for all datasets, the other methods also attained comparable recall only except CIDR, where it means that all the DEGs identified through the true cell-type labels are also covered by the DEGs determined by the predicted clustering labels (Figure 4a). Although the performance gap is not clear, except the Romanov data, SICLEN still achieved higher or comparable recall for other datasets. Interestingly, CIDR attained the smaller recall for Romanov, Baron_h1, Baron_h3, Baron_m1, and Baron_m2 datasets. One plausible explanation for the low recall achieved by CIDR is that the clustering labels obtained by CIDR may not be effective to predict the DEGs so that it results relatively the smaller number of DEGs compared to the other methods.

Next, although DEGs obtained by the predicted clustering labels result the high agreement with the true DEGs, if it includes several incorrect DEGs, it can mislead to understand the core insights in complex biological mechanisms. To verify the reliability of the predicted DEGs, we also assessed the precision for DEGs derived by each clustering label. Although SC3 and Seurat recorded the higher recall, their precisions are clearly smaller than SICLEN for the most datasets (Figure 4b). It means that the DEGs identified the clustering labels for SC3 and Seurat can have a larger number of incorrect DEGs. Additionally, SIMLR and CIDR recorded the smaller precision for the most datasets. However, the DEGs evaluated through the clustering labels by SICLEN still attained the higher precision then the other cutting-edge algorithms, where it means that the DEGs identified through the clustering labels of SICLEN includes the smallest number of incorrect DEGs.

Finally, we verified that SICLEN clearly achieved higher F-score for Usoskin, Kolod., Xin, Baron_m1, and Baron_m2 (Figure 4c). Additionally, SICLEN showed the comparable F-scores for the other datasets. In fact, all algorithms showed the similar F-scores for Darmanis, Klein, and Baron_h4 datasest. These results provide the strong evidence that the clustering labels produced by SICLEN is highly consistent with the true cell-type labels and it shows the effectiveness of the proposed single-cell clustering algorithm in applications of downstream single-cell analysis pipelines.

### 3.4. Improved Single-Cell Sequencing Imputation through an Accurate Clustering

Single-cell sequencing techniques have been gaining importance in biomedical research as it can profile cell specific gene expressions, where it is a distinctive advantage compared to a next-generation sequencing. Compared with the next-generation sequencing, since the single-cell sequencing protocols use the small amount of mRNA samples to profile relative gene expressions for individual cells, it is also possible that the expressed transcripts can be completely missed during reverse transcription steps, where it induces excessive zeros called the dropout events. To compensate these missing values, several imputation algorithms have been proposed [38,39,40,41,42]. Among these methods, scImpute [40] can take advantage of the cell types to infer the missing expression values in a single-cell sequencing, i.e., it can leverage the prior knowledge for the cell types so that it predicts the missing gene expressions based on the gene expression of cells that potentially belong to the same cell type.

To verify the compatibility of the proposed single-cell clustering algorithm in other single-cell analysis algorithms such as imputation methods, we supposed that if the predicted clustering labels can yield the high level of accordance with the true cell-type labels, the imputation results of scImpute based on the true cell type and predicted clustering labels can also achieve the higher degree of consensuses. To verify the consistency of the imputation results based on the true and predicted labels, we defined the normalized error for the gene expression values such that $\frac{\sum_{i\in D}^{}\sum_{\forall j}^{}\left|x_{i,j}-{\hat{x}}_{i,j}\right|}{\left|N\right|\cdot \left|D\right|}$, where *N* is the number of cells, and D is the set of DEGs identified by the group comparison through MAST, $x_{i,j}$ is the log-transformed gene expression for the *i*-th gene and *j*-th cell, where it is obtained by scImpute based on the true cell-type labels, ${\hat{x}}_{i,j}$ is the log-transformed gene expression for the *i*-th gene and *j*-th cell, where it obtained by scImpute using the predicted clustering labels. Among five algorithms, SICLEN showed the smallest normalized errors for the Darmanis, Usoskin, Kolod., Xin, Klein, and Baron_h3 datasets (Table 3), i.e., the imputation results through the predicted clustering labels derived by SICLEN showed the minimum differences to the imputation results using the true cell-type labels. As with the proposed algorithm, although CIDR also includes the noise reduction process in the main part of the algorithm, it showed relatively higher normalized error than that of SICLEN. Although SC3, Seurat, and SIMLR do not have a noise reduction component in the algorithm, their normalized error is smaller than that of CIDR. Through the result, we confirmed the powerful compatibility of the proposed algorithm because it showed the highest agreement with the imputation results using true cell-type labels.

**Figure 5:**
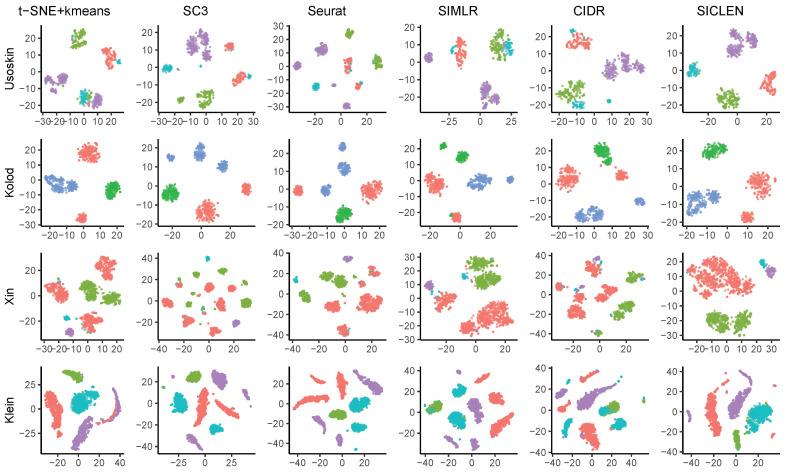
Low-dimensional visualization of the selected datasets. To visualize, we first reduce the zero-inflated noise through scImpute based on the true and predicted labels. Then, we obtain the low-dimensional representation through t-SNE.

Additionally, we compared the low-dimensional visualization of the imputation results through t-SNE. We obtained the two-dimensional coordinates for each cell using t-SNE and highlighted their labels based on the true cell types. The low-dimensional visualization through the imputation results using the predicted labels by SC3 and Seurat can divide the same cell types into multiple small clusters (Figure 5). Since SC3 and Seurat can overestimate the number of clusters, it can make multiple small clusters even though it is originally in the same types. In fact, if one can use the imputation and visualization results without the true cell-type labels (i.e., without the color labeling in the figure), it may have a risk to yield incorrect analysis results such as the number of clusters (i.e., cell types). Additionally, for the Usoskin, Xin, and Klein datasets, SIMLR and CIDR collect the different cell types into a single cluster. However, SICLEN can yield clear visualization for various cell types, where it can collect cells with the same types into a same cluster and separate cells with different cell types into different groups. Overall, the imputation results based on the clustering labels predicted by SICLEN can yield the clear separation for the different cell types and the tight collection for the same cell types. Based on the results, we verified that the predicted clustering labels obtained by SICLEN is relatively confident than the other algorithms, and it has a great compatibility to other single-cell analysis algorithms.

## 4. Discussion

We propose a novel single-cell clustering algorithm based on the effective noise reduction through the ensemble similarity network. First, we identify the set of the potential feature genes that can have a high probability to be the marker genes for each cell type. Based on the multiple random gene sampling from the set, we obtain the multiple cell-to-cell similarity measurements through Pearson correlation and construct the ensemble similarity network by inserting edges between cells if they achieve consistently high similarity based on different similarity estimations. Then, we adopt a random walk with restart approach to reduce the zero-inflated noise in the single-cell sequencing data. Finally, we drive the accurate single-cell clusters based on the iterative merging process of small but highly consistent single-cell clusters obtained by a *K*-means clustering algorithm. Through a comprehensive evaluation using real-world single-cell sequencing datasets, we demonstrate the effectiveness of the proposed single-cell clustering algorithm by showing the accuracy of clustering results, its potential for a downstream biological analysis, and flexibility to other single-cell analysis algorithms.

One of the major contributions of the proposed single-cell clustering algorithm is that the proposed method can avoid the complex optimal feature gene selection problem. Although a performance of the most single-cell clustering algorithms highly depends on the selection of the feature genes, many single-cell clustering algorithms overlook the importance of the optimal feature gene selection problem or they simply select a single set of genes to yield the final clustering results, where it is still not proved that the selected set is optimal to yield the best clustering results. However, while achieving a reliable clustering result, the proposed algorithm can avoid the optimal feature selection problem based on the multiple similarity estimates through a random gene sampling that can derive the robust estimation of the cell-to-cell similarity. In fact, although we cannot claim that the estimated cell-to-cell similarity is optimal, it still results accurate and reliable clustering results based on our experimental validations. Next, another contribution of the proposed work is deriving a tailored method to reduce the zero-inflated noise in a single-cell sequencing data. Although the artificial noise can lead to negative effects on single-cell clustering results, most of the state-of-the-art single-cell clustering algorithms do not have a noise reduction component. In the proposed method, the noise reduction module based on a random walk with restart framework can effectively reduce the noise and enhance the reliability of a single-cell sequencing data, where it can ultimately yield an accurate single-cell clustering. Furthermore, it also has a great potential to motivate other preprocessing methods for a single-cell sequencing data. One advantage of the proposed method is that it has a high flexibility to existing single-cell analysis pipeline because it does not change the dimension (i.e., the number of genes and cells) of a single-cell sequencing data. Moreover, since it does not require a prior knowledge such as marker genes for each cell type and the true number of clusters, it is quite practical and versatile to apply other single-cell analysis algorithms.

Although the proposed single-cell clustering algorithm have distinctive advantages over the cutting-edge algorithms, there are inevitable limitations. First, although the proposed method can estimate the true number of clusters in a single-cell sequencing data, since it is not a perfect method and there is an inherent error. Moreover, since one cell type can be divided into multiple subtypes, it is a still challenging open problem to derive an accurate method to simultaneously estimate the true number of cell types and subtypes through a precise identification of a hierarchical structure for different cell types. Second, although reducing zero-inflated noise through a random walk with restart is one of the distinctive advantages of the propose method over other state-of-the-art algorithms, if a single-cell sequencing data includes a larger number of dropout events, the missing gene expression may not be recovered based on the current approach, i.e., if all genes in a specific cell type are completely missed across the majority of cells because of severe dropout events, these missing values cannot be inferred based on the current approach because it requires the gene expression from multiple cells with high similarity. In fact, this is a common problem for most noise reduction methods using a cell-to-cell similarity. To resolve the problem, it can be necessary to derive an effective method to simultaneously employ both a cell-to-cell similarity and gene-to-gene correspondence. Next, although the proposed method can only predict the clustering labels, to unveil key biological mechanisms, it requires in-depth analysis such as constructing a gene regulatory network, pseudo-time ordering and inferring the origin of cells in the tissue samples. To fill out these gaps, the desirable direction for the future work is developing comprehensive downstream analysis pipeline by integrating additional biological analysis modules such as identifying differentially expressed genes, effective low-dimensional visualization methods, inferring cell-type-specific gene regulatory networks, and pseudo-time ordering based on the clustering results. Finally, supporting parallel computing to reduce the running time of the algorithm is also necessary to accelerate an in-depth analysis of a large-scale single-cell sequencing data and providing a user-friendly UX (user experience) or cloud platform can be the appropriate future work.

## Figures and Tables

**Table 1:** Basic statistics of the single-cell sequencing datasets.

Dataset	# Genes	# Cells	# Clusters	Source	Accession
Darmanis [19]	21,517	420	8	Human brain	GSE67835
Usoskin [21]	19,534	622	4	Mouse sensory neurons	GSE59739
Kolod. [22]	10,684	704	3	Mouse embryo stem cells	E-MTAB-2600
Romanov [23]	21,143	2881	7	Mouse hypothalamus	GSE74672
Xin [24]	33,584	1492	4	Human pancreas	GSE81608
Klein [2]	24,047	2717	4	Mouse embryo stem cells	GSE65525
Baron_h1 [25]	15,452	1622	6	Human pancreas	GSE84133
Baron_h2 [25]	15,810	1562	6	Human pancreas	GSE84133
Baron_h3 [25]	16,386	3333	6	Human pancreas	GSE84133
Baron_h4 [25]	15,285	1225	6	Human pancreas	GSE84133
Baron_m1 [25]	13,757	687	5	Mouse pancreas	GSE84133
Baron_m2 [25]	14,105	932	5	Mouse pancreas	GSE84133

**Table 2:** The number of differentially expressed genes for each datasets. Please note that the DEGs (ground truth) are identified through the raw data and true cell-type labels.

Datasets	# DEGs	Datasets	# DEGs
Darmanis	5828	Baron_h1	695
Usoskin	2730	Baron_h2	494
Kolod.	3278	Baron_h3	566
Romanov	1842	Baron_h4	652
Xin	3499	Baron_m1	399
Klein	4819	Baron_m2	527

**Table 3:** Normalized error for the noise reduced gene expressions for DEGs. DEGs are identified through MAST and zero-inflated noise is reduced through scImpute. Please note that the best performers are highlighted as a boldface font.

Method	Darmanis	Usoskin	Kolod.	Xin	Klein	Romanov	Baron_h1	Baron_h2	Baron_h3	Baron_h4	Baron_m1	Baron_m2	Total
SC3	0.633	0.608	0.127	0.593	0.379	0.721	0.564	0.631	0.646	0.537	**0.799**	0.831	7.068
Seurat	0.566	0.636	0.133	0.405	0.400	0.751	0.489	0.581	0.611	0.426	0.850	0.846	6.693
SIMLR	0.729	0.605	0.163	0.344	0.387	**0.665**	**0.479**	**0.557**	0.551	**0.376**	0.873	**0.788**	6.515
CIDR	0.605	0.610	0.189	0.364	0.575	0.748	0.721	0.848	0.611	0.654	1.369	0.869	8.164
SICLEN	**0.485**	**0.603**	**0.015**	**0.314**	**0.354**	0.769	0.503	0.597	**0.535**	0.421	0.833	0.869	**6.299**

## Data Availability

The source code of the proposed method is freely available at https://github.com/jeonglab/SICLEN.

## Supplementary Materials

The following are available online at https://www.mdpi.com/article/10.3390/genes12111670/s1.
