# Supplementary material for: Accurate Single-Cell Clustering through Ensemble Similarity Learning
Source: Genes (Basel). 2021 Oct 22;12(11):1670. doi: 10.3390/genes12111670 (PMC8623803; doi:10.3390/genes12111670)
Supplement: Supplementary file 1 [file genes-12-01670-s001.zip › genes-1329044-supplementary.pdf]

Supplementary Material for Accurate single-cell clustering through ensemble similarity learning

Hyundoo Jeong[1], Sungtae Shin[2], and Hong Gi Yeom[3]*
[1] Department of Mechatronics Engineering, Incheon National University, Incheon, 22012, R.O.Korea
[2] Department of Mechanical Engineering, Dong-A University, Busan 49315, R.O.Korea
[3] Department of Electronics Engineering, Chosun University, Gwangju 61452, R.O.Korea

*honggi@chosun.ac.kr

S1 Sensitivity analysis of free parameters

We assessed the performance of the proposed method based on different free parameter settings. The proposed single-cell clustering algorithm has four free parameters as follows:

- `nens`: The number of iteration for the ensemble similarity learning. That is, `nens` indicates the number of similarity measurements using different feature sets. Although the larger number could yield the more reliable similarity learning, it can also require longer computation time. We set the 20 as the default setting.
- `knum` : The number of neighboring nodes for constructing KNN (K-nearest neighboring) network. We set the 30 as the default setting.
- `alpha` : The restarting probability of the random walker. If alpha is equal to zero, the random walker will not restart and stay the initial location. Hence, the zero-inflated noise will not be removed. This is a key parameter and the sweet spot typically ranges from 0.5-0.8. We set the 0.7 as the default setting.
- `npc` : The number of principal components to estimate the cell-to-cell similarities. Based on our experiment, 10 PCs are enough for the most single-cell sequencing datasets. We set the 10 as the default setting.

To verify the sensitivity of parameter settings, we employed the default parameter settings and evaluated the performance changes by changing only one free parameters.

S1.1 Sensitivity analysis for the different numbers of ensemble learning

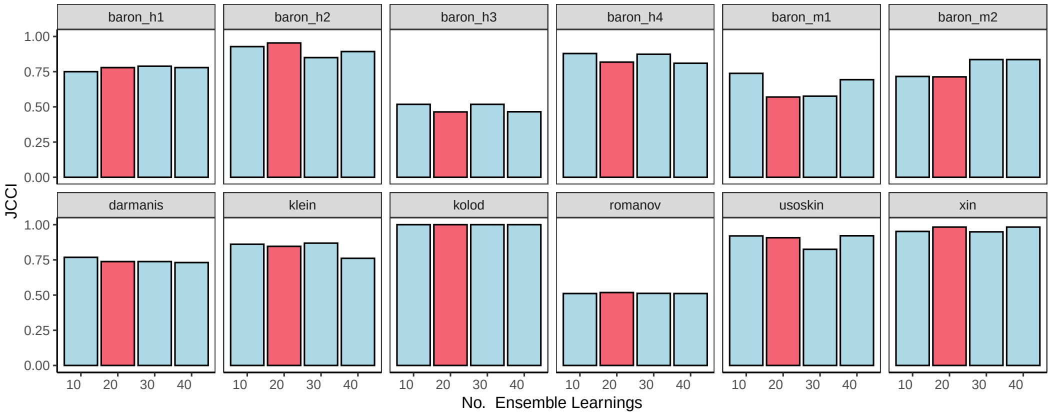

(a) Jaccard index for different number of similarity measurements.

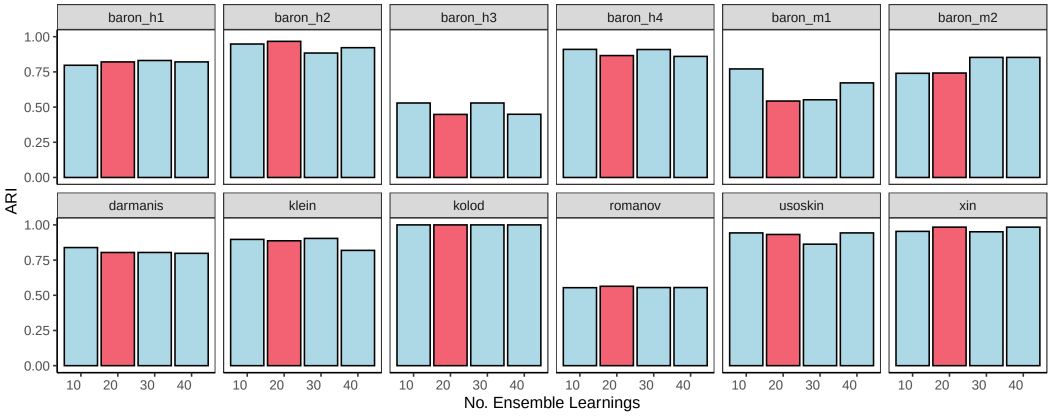

(b) Adjusted rand index for different number of similarity measurements.

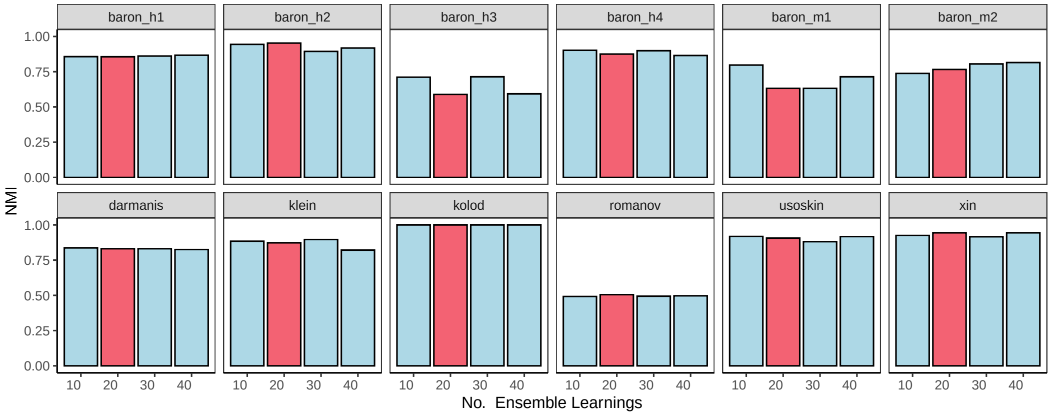

(c) Normalized mutual information for different number of similarity measurements.

Figure S1: Sensitivity analysis for the different numbers of ensemble learning. Note that the red bar indicates the default parameter setting for the proposed method.

S1.2 Sensitivity analysis for the different numbers of neighboring nodes

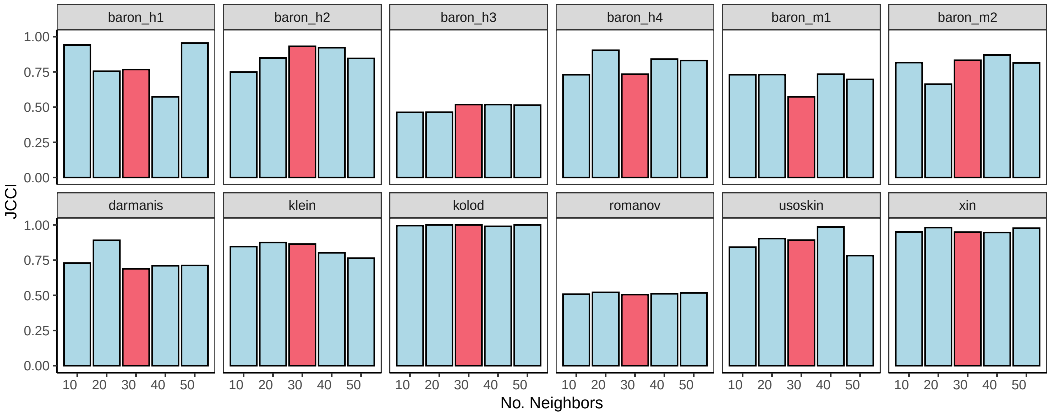

(a) Jaccard index for different number of neighboring nodes.

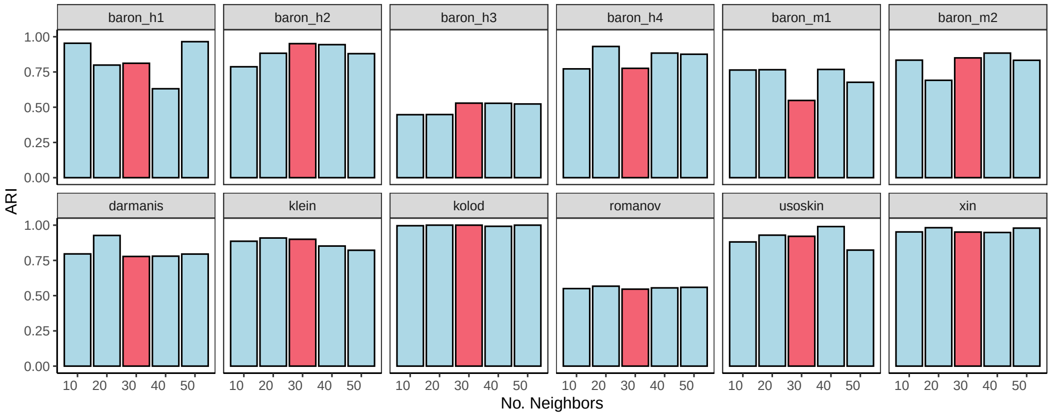

(b) Adjusted rand index for different number of neighboring nodes.

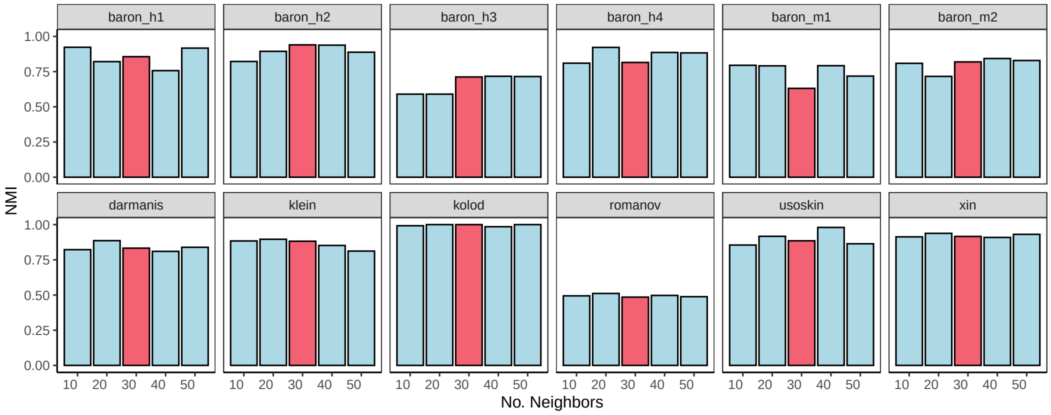

(c) Normalized mutual information for different number of neighboring nodes.

Figure S2: Sensitivity analysis for the different numbers of neighboring nodes. Note that the red bar indicates the default parameter for the proposed method.

S1.3 Sensitivity analysis for the different values of α

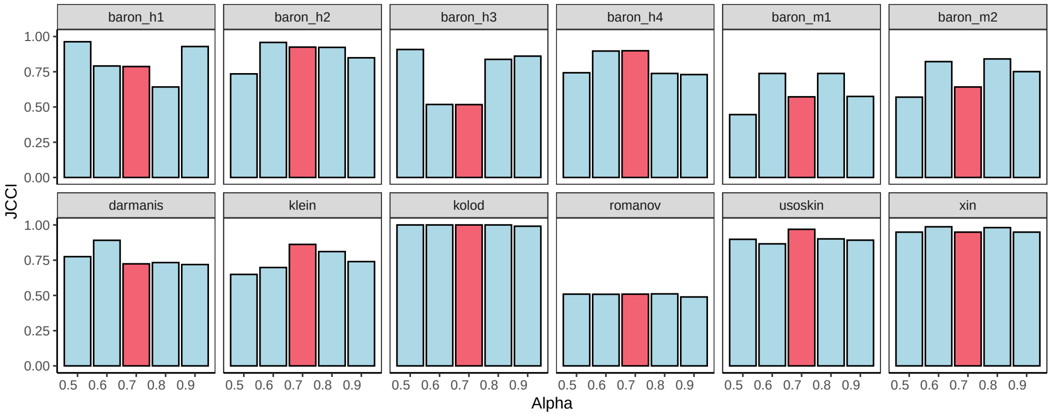

(a) Jaccard index for different value of α.

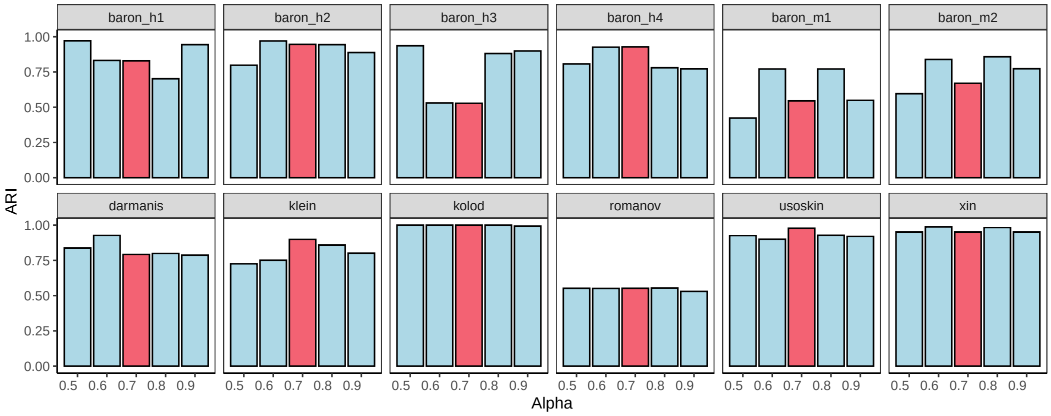

(b) Adjusted rand index for different value of α.

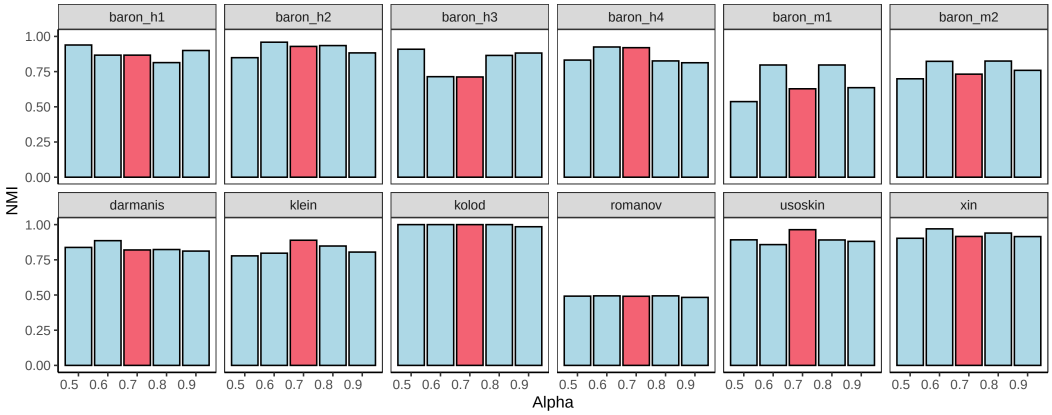

(c) Normalized mutual information for different value of α.

Figure S3: Sensitivity analysis for the different values of α. Note that the red bar indicates the default parameter for the proposed method.

S1.4 Sensitivity analysis for the different number of principal components

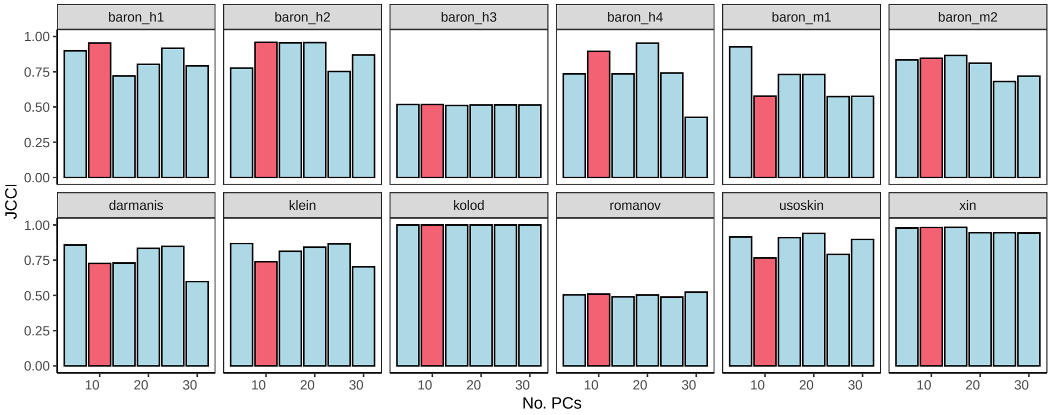

(a) Jaccard index for for different number of PCs.

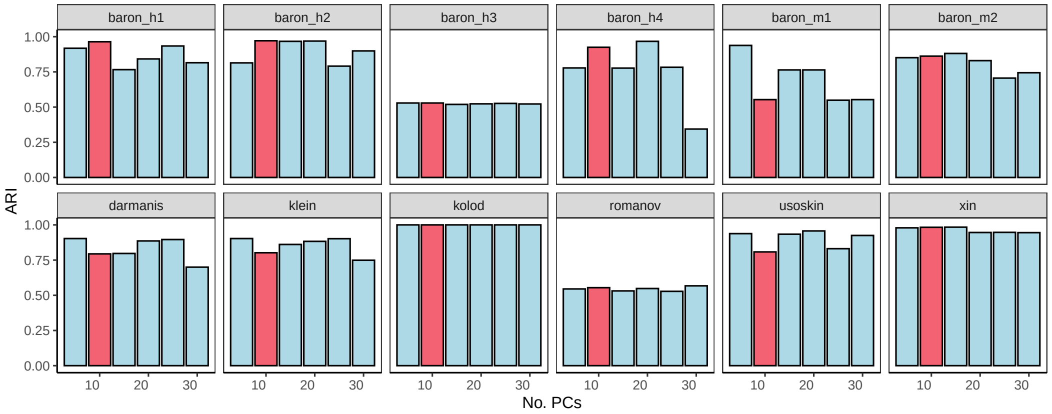

(b) Adjusted rand index for for different number of PCs.

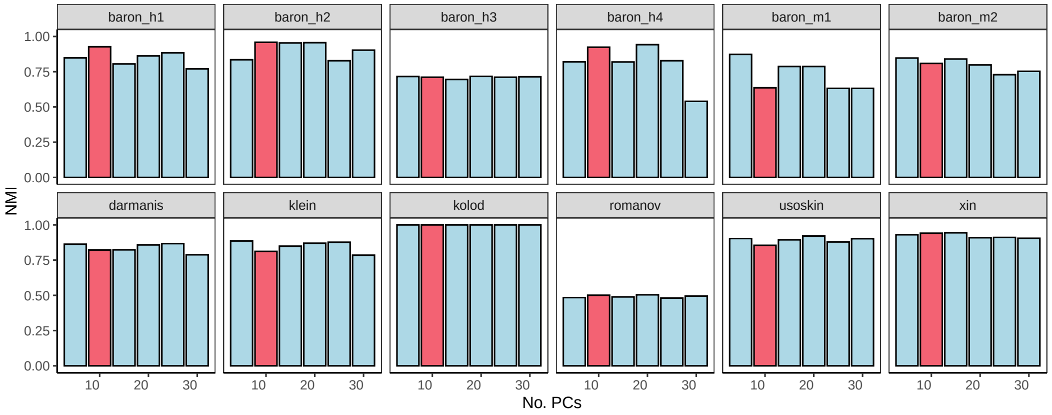

(c) Normalized mutual information for for different number of PCs.

Figure S4: Sensitivity analysis for the different number of principal components. Note that the red bar indicates the default parameter for the proposed method.
